# IS HIV SHORT-SIGHTED? INSIGHTS FROM A MULTISTRAIN NESTED MODEL

**DOI:** 10.1111/evo.12166

**Published:** 2013-06-13

**Authors:** Katrina A Lythgoe, Lorenzo Pellis, Christophe Fraser

**Affiliations:** 1 Department of Infectious Disease Epidemiology, School of Public Health, Imperial College LondonSt. Mary's Campus, London, W2 1PG, United Kingdom; 2 E-mail: k.lythgoe@imperial.ac.uk; 3 Mathematics InstituteZeeman Building, University of WarwickCoventry, CV4 7AL, United Kingdom

**Keywords:** Epidemiology, evolution, nested model, pathogen, transmission-virulence trade-off, virulence

## Abstract

An important component of pathogen evolution at the population level is evolution within hosts. Unless evolution within hosts is very slow compared to the duration of infection, the composition of pathogen genotypes within a host is likely to change during the course of an infection, thus altering the composition of genotypes available for transmission as infection progresses. We develop a nested modeling approach that allows us to follow the evolution of pathogens at the epidemiological level by explicitly considering within-host evolutionary dynamics of multiple competing strains and the timing of transmission. We use the framework to investigate the impact of short-sighted within-host evolution on the evolution of virulence of human immunodeficiency virus (HIV), and find that the topology of the within-host adaptive landscape determines how virulence evolves at the epidemiological level. If viral reproduction rates increase significantly during the course of infection, the viral population will evolve a high level of virulence even though this will reduce the transmission potential of the virus. However, if reproduction rates increase more modestly, as data suggest, our model predicts that HIV virulence will be only marginally higher than the level that maximizes the transmission potential of the virus.

Understanding how pathogens evolve at the epidemiological level is vital if we are going to accurately assess how epidemics and pandemics are likely to progress, and what the consequences of biomedical and other interventions are likely to be. Important components of pathogen evolution at the population level are the ecological and evolutionary processes that occur during the course of an infection. As a consequence, pathogens can face conflicting evolutionary pressures because traits that maximize the within-host fitness of a pathogen strain might reduce its between-host fitness. This conflict will be particularly strong when multiple pathogen strains persist in a single host, either due to infection by multiple strains or by the generation of multiple strains through mutation. Here, we develop a nested modeling framework that allows us to follow the evolution of pathogens at the epidemiological level, and find equilibrium values, by explicitly considering the within-host evolutionary dynamics of multiple competing strains and the timing of transmission. We use the framework to assess the impact of within-host processes on the evolution of virulence of human immunodeficiency virus (HIV) at the epidemiological level, although the approach could be applied to a number of host–pathogen systems.

The relatively stable set-point viral load (SPVL) of HIV observed during chronic asymptomatic infection is a commonly used proxy for virulence (Müller et al. 2011). A high SPVL increases the probability that virus will be transmitted, but also hastens the onset of AIDS and eventual death, thus reducing the period during which the virus can be transmitted (Mellors et al. 1996; De Wolf et al. 1997; Korenromp et al. 2009). SPVL is at least partly heritable from infection to infection (Tang et al. 2004; Alizon et al. 2010; Hecht et al. 2010; Hollingsworth et al. 2010; van der Kuyl et al. 2010), and it has recently been shown that HIV seems to have evolved an intermediate level of virulence that maximizes the number of potential new infections from a single infection, known as its transmission potential (Fraser et al. 2007; Shirreff et al. 2011), thus maximizing the between-host fitness of the virus. With a higher level of virulence, the virus is more likely to be transmitted while the infection lasts, but death due to AIDS will be swifter, resulting in fewer onward infections during the lifetime of the infected individual. With a lower level of virulence the host will live longer, but the rate of onward transmission will be lowered, again reducing the number of onward infections during the lifetime of the infected individual.

However, the evolution of an intermediate level of virulence that maximizes transmission potential sits uncomfortably with the concept of short-sighted evolution (Levin and Bull 1994; Frank 2012). During the course of long-term infections we should expect strains with a competitive advantage to sweep through the within-host population if and when they arise, regardless of whether this reduces the transmission potential of the current or subsequent infections. Evolution is “short-sighted” because what is good for the virus in the short-term within the host is not necessarily what is good for the virus in the longer-term at the epidemiological level. This is analogous to the concept of the tragedy of the commons seen in models of social evolution (Rankin et al. 2007).

There is good reason to believe that the evolution of HIV should be short-sighted. Infection with a strain of HIV that has a high (low) replicative capacity is likely to result in an infection with a high (low) viral load (Quiñones-Mateu et al. 2000; Trkola et al. 2003; Daar et al. 2005; Joos et al. 2005; Kouyos et al. 2011). In other words, the replicative capacity of a viral strain is correlated with virulence. HIV can evolve extremely quickly during the course of infection (Shankarappa et al. 1999; Lemey et al. 2007), on the face of it giving the virus ample opportunity to produce strains with a high replicative capacity and for these to sweep through the within-host population. Evidence suggests that the replicative capacity of HIV does indeed tend to increase during the course of an infection (Troyer et al. 2005; Kouyos et al. 2011). As a consequence, if within-host fitness is correlated with virulence, we might expect the virulence of HIV to be relatively high as a result of short-sighted evolution, even if this is not the best strategy for the virus at the epidemiological level.

To better understand the conflicting evolutionary pressures influencing the evolution of HIV virulence, we have constructed a nested modeling framework that integrates within-host evolutionary dynamics and between-host dynamics, and that allows a large number of strains to coexist within a host at any one time. A growing number of models have been developed enabling us to link within-host and between-host dynamics (examples include: Sasaki and Iwasa 1991; Gilchrist et al. 2002; André and Gandon 2006; Gilchrist and Coombs 2006; Coombs et al. 2007; Alizon and van Baalen 2008; Luciani and Alizon 2009; Feng et al. 2011; Saenz and Bonhoeffer 2013), but apart from the individual-based HCV simulation study of Luciani and Alizon (2009), none have considered more than two strains coexisting within a host at any one time. Incorporating multiple strains into our models, when they affect phenotype and when they are likely to coexist within hosts, is important because otherwise it is impossible to adequately assess the consequences of within-host ecological and evolutionary processes on the epidemiological dynamics of pathogens. Here, we explicitly incorporate the evolution of pathogens through the course of infection, allowing for differential transmission of pathogen strains depending on their frequency within the host at the time of transmission, the intensity of the infection, and the inherent transmissibility of the strain. Unlike other nested models, we model the within-host dynamics using a quasi-species approximation, meaning that the frequency of the different strains within the host are determined by their reproduction rates and by the probability of mutation from one strain to another at the time of replication. The frequencies are then multiplied by empirically determined infectivity profiles to express the viral load during the course of infection, and the duration of infection as a function of the virulence of the infecting strain (Fraser et al. 2007; Shirreff et al. 2011). This allows for very efficient computation of the within-host dynamics. However, if desired, a more mechanistic model could be used instead.

Crucially, we find that by changing the shape of the within-host adaptive landscape and the intensity of within-host and between-host competition, we can reach qualitatively different predictions, including whether one or multiple strains persist within the viral population, and whether the virus evolves toward low, intermediate, or high levels of virulence at the population level. Indeed, in some cases the prevalence gets to such low levels that the viral population effectively drives itself to extinction. In all cases, as the intensity of within-host competition increases, the fitness of the viral population at the epidemiological level, as measured by the basic reproduction number *R*_0_, decreases.

Data suggest that the replicative capacity of HIV increases during the course of infections, but by a relatively modest amount compared to the variance in replicative capacities observed across patients (Troyer et al. 2005; Kouyos et al. 2011). In this situation, our model predicts that between-host processes will overshadow within-host processes and therefore HIV will evolve a level of virulence that is similar to the level that maximizes *R*_0_. If interpreted in terms of within- versus between-group selection (where here a group is the collection of viruses within an individual), the result agrees with our intuition from standard theory. That is, when variation among groups is much larger than variation within groups, selection at the between-group level will overshadow selection at the within-group level (see Frank 2012 for a further details).

Although here we apply our framework to consider the problem of virulence in HIV, it is a general framework that could be applied to a large number of host–pathogen systems in which individual hosts are coinfected by multiple strains, or where new strains arise frequently as a consequence of mutation. Importantly, this modeling framework is extremely flexible. For example, it can be used in conjunction with an explicit model of within-host evolution, or as implemented here, it can incorporate a more generic quasi-species model of within-host evolution combined with empirically determined infectivity profiles.

## Methods

We construct our nested model by linking the within-host evolutionary dynamics of HIV with between-host dynamics, describing the spread of the virus in an exposed human population. The between-host modeling framework we use is based on the well-developed theory of multitype epidemic models (Diekmann and Heesterbeek 2000). In this framework, individuals are divided into types based on their epidemiological characteristics. We assume that once a host is infected the course of infection is only driven by within-host dynamics and is not influenced by other factors. This is a common assumption when dealing with microparasites, and its main consequence is that we can rely on standard epidemiological theory for the spread of the infection at the between-host level. However, it also means that we ignore effects such as superinfection. This assumption should be revisited once the dynamics of superinfection are more clearly understood. For example, it has recently been shown that superinfection is much more common than was previously thought, although it is unclear how this influences the course of infection (Redd et al. 2012b).

### WITHIN-HOST MODEL

It is now well known that most new heterosexual HIV infections are established by a single virion (Keele et al. 2008), and therefore for simplicity we assume that all new infections are established from a single viral strain. We also assume that host factors do not influence the course of the infection, so that all susceptible individuals are identical. Because only within-host factors influence infection, the course of infection is uniquely determined by the strain initiating it, and therefore we can identify infected individuals by their type, where a type-*j* individual is someone initially infected with strain *j*. The assumption of infection by a single strain and identical susceptible hosts can easily be relaxed at the price of increasing the number of types.

Because of mutation and subsequent competition within individuals, someone infected by strain *j* can infect a susceptible person with strain *i*. In a fully susceptible population, a type-*j* individual will generate type-*i* individuals through transmission at a time-dependent rate β*_ij_*(τ), where τ is the time since the type-*j* individual was infected (τ is often referred to as the age of infection).

The within-host component of the model is used to characterize the strain-specific infectivity profile, β*_ij_*(τ) for all infection types.

The strain-specific infectivity profile of a type-*j* infection could be determined using a mechanistic within-host model, describing the interaction of the virus with the host's immune system (e.g., Coombs et al. 2007). However, simple competition models cannot reproduce the complex profiles of time-varying infectivity characteristic of HIV, and therefore we use a more pragmatic approach. By using available data, we define an overall time-varying infectivity profile α*_j_*(τ) for a type-*j* individual, and model the change in frequencies of each strain, *x_ij_*(τ), within each type of host using the reproduction–mutation quasi-species equation (Nowak 2006). Here *x_ij_*(τ) represents the frequency of strain *i* in a type-*j* host at time τ since infection. We finally assume that strains can differ relative to each other in how efficiently they are transmitted between hosts, and denote the relative between-host transmissibility of strain *i* by *G_i_*. Combining all these elements, we obtain the general equation for the strain-specific infectivity profile:


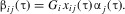
(1)

To define the overall infectivity profile, α*_j_*(τ), for a type-*j* individual, we follow Shirreff et al. 2011 (see also Saenz and Bonhoeffer 2013). The overall infectivity profile of HIV is assumed to have three stages: primary, asymptomatic, and AIDS. The duration of infection and the infectivity of the primary and AIDS stages are assumed to be equal for all infections, but the more virulent the infecting strain, the greater the infectivity and the shorter the duration of the asymptomatic stage, and therefore the shorter the duration of the entire infection, *T_j_* (see fig. 2 of Fraser et al. 2007 and Shirreff et al. 2011 for further details and the precise formalization).

To calculate the frequency of strain *i* in a type*-j* host, *x_ij_*(τ), we use the reproduction-mutation quasi-species equation, as follows: let **y** = (*y_i_*) be the (column) vector of the number of virions of each strain within a host in an unbounded reproduction–mutation system. Also, let *M* = (*m_ij_*) be the mutation matrix, where *m_ij_* is the probability that the progeny of a strain-*j* virion is a strain-*i* virion. Next, let *g_i_* be the reproduction rate of strain *i*. Finally, let *Q* = (*q_ij_*) = (*m_ij_g_j_*) be the reproduction–mutation matrix. Then the unbounded reproduction–mutation system is described by the equation:


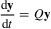
(2)

with initial condition 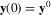 and solution 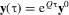, where we have made use of matrix exponentiation. Denoting the total number of virions by 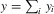, the system for the frequencies 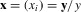 is given by the quasi-species equation:


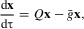
(3)

where 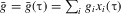. The second term in equation (3) ensures that the strain frequencies always sum to 1. Given the initial condition 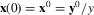, the *i*th element of the solution of equation (3), 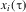, can be written in the form (Domingo et al. 2008):


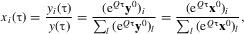
(4)

where 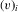 represents the *i*th element of vector *v*. The last equation follows because the solution does not change if the initial condition is multiplied by any arbitrary constant.

So far, we have let the viral population grow unbounded. However, if we assume that virion death, for example due to the immune system, limits the viral load, and that the death rate is strain independent, the above equation still holds. This is, admittedly, a very strong assumption, but is essential for analytical tractability. For the same reason of tractability, we also assume that the reproduction rates and mutation probabilities of the different strains remain constant throughout an infection. The death rate instead is allowed to vary during the course of the infection, and this is assumed to happen in such a way that the total viral load becomes consistent with the required overall infectivity profile.

If the infection starts with a single virion of strain *j*, then we can write 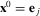, where 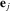 denotes the column vector with all 0 elements, except for a single 1 in position *j*. It follows that


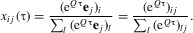
(5)

Because 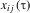 is the frequency of strain *i* virions in a type-*j* host at time τ since infection, (5) can be substituted into equation (1) to calculate the strain-specific infectivity profile.

### BETWEEN-HOST MODEL

We next describe the between-host model. All individuals have a natural mortality rate μ and, in addition, type-*j* individuals are assumed to die at time 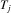 after infection, assuming they have not already succumbed to natural mortality. As described in the previous section, the duration of infection is only affected by the duration of asymptomatic infection, which in turn is determined by the virulence of the infecting strain (Shirreff et al. 2011). Denoting the time elapsed since the beginning of the epidemic by *t*, the force of infection of strain *i* at time *t* due to a type-*j* individual infected at time 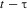 is 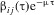 for 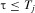 and 0 for 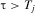. The factor 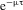 represents the fraction of individuals infected at time 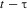, which have survived up to time *t*.

Assuming random mixing, if the population is not fully susceptible, only a fraction 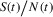 of infectious attempts will result in a real infection, where 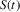 is the number of susceptible individuals at time *t* and 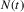 is the total population size at time *t*. Denoting the incidence of type-*i* cases at time *t* by 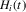, where incidence is defined as the rate of new infections, and assuming that individuals enter the exposed population with constant overall birth rate *B*, the epidemiological dynamics are given by


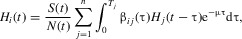
6 1


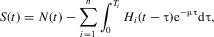
6 2


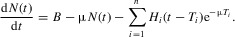
6 3

The underlying epidemiological model used here is a susceptible-infected (SI) model with demography. The quantity 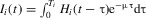 appearing in the second line of equation (6.2) represents the total number of type-*i* individuals at time *t*. Using a star to denote quantities at equilibrium, we find that


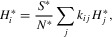
7 1


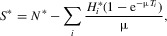
7 2


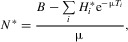
7 3

where 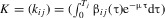 is the so-called next-generation matrix (Diekmann and Heesterbeek 2000). Each element 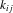 represents the average number of type-*i* infections generated in a fully susceptible population by a type-*j* individual during their entire infectious life. Standard epidemiological theory (Diekmann and Heesterbeek 2000) allows fast analytical computation of all quantities at equilibrium, thanks to the use of the next-generation matrix as follows. Perron–Frobenius theory of positive matrices assures that *K* has a unique (real) dominant eigenvalue, which represents the correct definition of the basic reproduction number *R*_0_ (see Diekmann and Heesterbeek 2000, ch. 5.1). Also, from equation (7.1), the vector 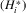 representing the incidence of each type at equilibrium is an eigenvector of *K* relative to *R*_0_. Furthermore, *R*_0_ represents the average number of new infections a typical infected individual generates in a fully susceptible population (see Diekmann and Heesterbeek 2000, p. 95, for the precise meaning of the word “typical”). At equilibrium, the number of infected individuals does not change, so that on average each infective must generate one new infection before dying. Therefore, the condition for equilibrium is


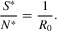
(8)

By denoting the total incidence at equilibrium by 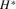, and the eigenvector of *K* relative to *R*_0_, normalized to have components summing to 1, by 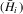, we have 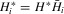. With some algebraic manipulation, we find that, at equilibrium:


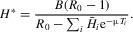
(9)

Because we can derive *R*_0_ and 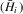 from *K* and 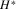 from equation (9), we can calculate all quantities at equilibrium from Equations (7.1)-(7.3). As already noted, here we have used an SI model with demography, as it applies to the case of HIV, but in principle any between-host model structure could be used, as long as it can be described using a next-generation matrix formalism.

## Results

We are interested in how within-host evolutionary dynamics affect the evolution of virulence at the epidemiological level. We start by considering the within-host dynamics of infection, and then look at the epidemiological dynamics, before finally analyzing how the system behaves at equilibrium.

### WITHIN-HOST DYNAMICS

We consider *n* strains, indexed with 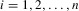, and we assume the higher the index of the strain initiating an infection, the higher the SPVL and therefore the more virulent the infection. Following Shirreff et al. (2011), we assume the viral loads of the strains are evenly distributed on a log scale, with infection by the least virulent strain resulting in a SPVL of 1 × 10^2^ viral particles per milliliter, and infection by the most virulent strain a SPVL of 1 × 10^7^ viral particles per milliliter. In addition, we define the within-host fitness of strain *i* as its reproduction rate 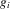 and assume that, for increasing *i*, such rates are evenly distributed on a linear scale, with the least virulent strain also having the lowest fitness. Therefore, within-host fitness and virulence are positively correlated. Here, 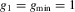 per day throughout, but the value of 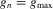 varies.

**Figure 1:**
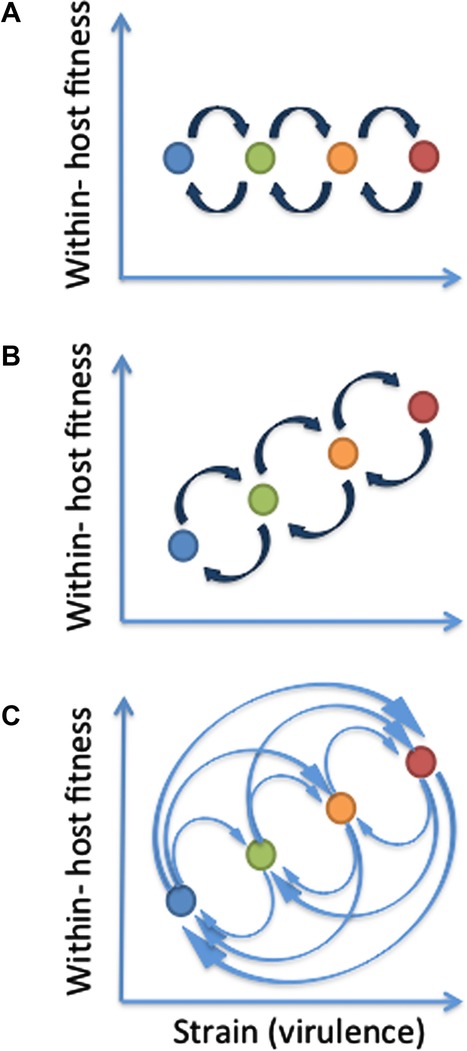
Representation of the three within-host fitness landscapes considered. (A) Flat fitness landscape. (B) Hill-climb fitness landscape. (C) Rugged fitness landscape. Note that, for the rugged landscape, lethal fitness valleys separate the strains (not shown) and therefore double mutations are required to move from one part of the landscape to another. Strains are represented by circles, and the higher the strain index, the more virulent the virus (strain 1, dark blue; strain 2, green; strain 3, orange; strain 4, red). The virulence of the strain initiating an infection determines the overall infectivity profile of the infection (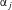) and the duration of the infection (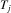). Dark blue arrows represent a mutation probability of 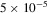 per replication. Light blue arrows represent a mutation probability of 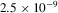 per replication.

The HIV within-host fitness landscape appears to be incredibly complex (Kouyos et al. 2012). To gain an understanding of the role the landscape has in our model, we consider three idealized within-host fitness landscapes (Fig. 1). The first is a flat fitness landscape where 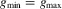, that is all strains have equal within host fitness, and where strain *i* mutates into strains 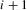 or 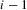 with probability 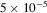 per replication (Mansky and Temin 1995; Gao et al. 2004). The second is a traditional hill-climb fitness landscape where the strains have different within-host reproduction rates and where strain *i* mutates into strains 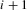 or 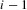 with probability 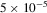 per replication. The third we refer to as a rugged landscape in which strains have different within-host reproduction rates, all have equal probability of mutating to any other strain, but where the strains are separated by a lethal fitness valley: only virions harboring a double mutation can cross the fitness valley, leading to a mutation probability between strains of 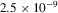 per replication. Because of the lethal fitness valleys separating the viable genotypes, this landscape can also be thought of as a “holey” fitness landscape that incorporates some features of the hill-climb fitness landscape.

**Figure 2:**
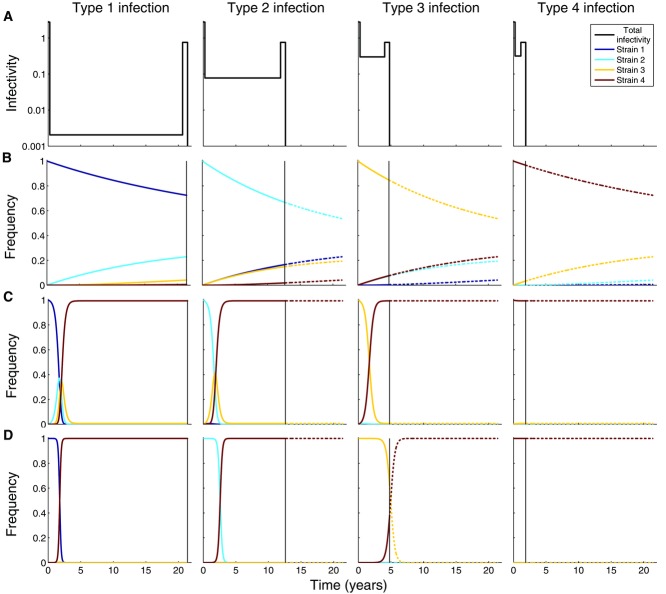
Within-host dynamics for the four-strain model for different within-host fitness landscapes. (A) Infectivity profile 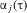 of type-*j* individuals (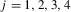). This is the rate (per year) at which new infections are made in a fully susceptible homogeneously mixing population during the course of infection. The type of an infected individual is defined by the strain initiating the infection. The least virulent strain is strain 1, and the most virulent is strain 4. The duration and infectiousness of the primary and AIDS stages of infection are the same for all infected individuals, but the more virulent the initiating strain, the shorter and more infectious is the asymptomatic stage of infection. (B) Relative frequencies of the four strains, by infection type, for a flat within-host fitness landscape. All strains have the same replicative capacity. (C) Relative frequencies of the four strains, by infection type, for a hill-climb fitness landscape. Strain 1 has the lowest reproduction rate and strain 4 has the highest reproduction rate (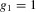 per day and 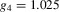 per day). (D) Relative frequencies of the four strains, by infection type, for a rugged fitness landscape. Strain 1 has the lowest reproduction rate and strain 4 has the highest reproduction rate (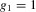 per day and 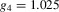 per day).

We can see from Figure 2A that individuals infected with a more virulent strain will be more infectious during asymptomatic infection, but that the duration of asymptomatic infection will also be shorter. Because the infectivity profile of the infection, 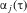, is only determined by the genotype of the infecting strain, all type-*i* infections have the same infectivity profile regardless of the shape of the within-host fitness landscape. If we assume a flat fitness landscape with four strains, the strain initiating the infection tends to dominate the within-host dynamics, although gradually the other strains reach appreciable frequencies as they approach mutation–selection balance (Fig. 2B). If the strains have unequal fitnesses at the within-host level, the strain with the highest within-host reproduction rate will increase in frequency as the infection progresses (Fig. 2C, D). In general, the larger the fitness difference between any pair of strains, the faster the fitter strain outcompetes the less fit strain. This is evident when we look at the rugged fitness landscape (Fig. 2D), where the fact that any strain can mutate in any other strain puts the fittest and the starting strains in direct competition with each other, resulting in faster dynamics when the starting strain reproduces relatively slowly (Fig. 2D). With a smooth hill-climb fitness landscape, the situation is more complex because strains that have a slow rate of reproduction cannot mutate directly into rapidly reproducing strains, but instead need to traverse the fitness landscape through the generation of all intermediate strains, and this process slows down the dynamics (Fig. 2C). Our results suggest that the effects of these two conflicting factors cancel each other out to a good approximation. Apart from when the starting strain is very unfit, the rugged fitness landscape exhibits slower within-host dynamics compared to the hill-climb landscape because of the reduced mutation rate between viable strains. For a fixed number of strains, widening their fitness range results in faster within-host dynamics. However, keeping the range fixed and increasing the number of strains reduces the fitness differences between adjacently numbered strains, and tends to slow down the within-host dynamics (not shown).

### EPIDEMIOLOGICAL DYNAMICS

Nesting the within-host model into the epidemiological model, we can see how the within-host dynamics influence the evolutionary epidemiology of the virus. The equations describing the dynamical population model (equations 6.1–6.3) were solved numerically using the basic Euler forward method and programmed independently in two mathematical packages, Mathematica (KL) and Matlab (LP), enabling us to cross-validate the results. In all simulations, the initial population size, *N*, is 10,000, and all individuals are susceptible except for one individual infected by the least virulent strain. A list of parameters and variables are given in Table1.

**Table 1:** Variables and parameters used in the model

Variables	Definition	Values
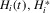	Incidence (defined as number of new cases per year), of type-*i* infections at time *t* and at equilibrium	
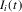	Total number of type-*i* individuals at time *t*	
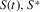	Number of susceptible hosts at time *t* and at equilibrium	
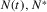	Total number of hosts at time *t* and at equilibrium	
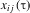	Frequency of strain *i* in a type-*j* individual at time τ since infection	
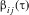	Strain-specific infectivity profile: the rate at which type-*j* individuals transmit strain *i* at time τ since infection in a fully susceptible population	
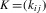	Next generation matrix. Each element 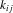	
*R*_0_	Basic reproduction rate of the viral population at equilibrium	
Parameters		
*B*	Rate at which individuals enter the exposed population	200 per year
μ	Host natural per-capita mortality rate	0.02 per year
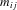	Probability of strain *j* mutating into strain *i* during replication	0.5×10^−5^ or 2.5×10^−9^
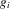	Within-host replication rate of strain *i*	Variable
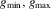	Minimum and maximum within host reproduction rates	1, variable
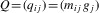	Reproduction-mutation matrix	Variable
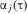	Overall infectivity profile of type-*j* individuals as a function of time τ since infection	Variable
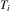	Duration of a type-*i* infection from time of infection to AIDS related death	Variable
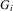	Relative between-host transmissibility of strain *i*	Variable
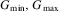	Relative minimum and maximum between-host transmissibility	1, 1 or 5

**Figure 3:**
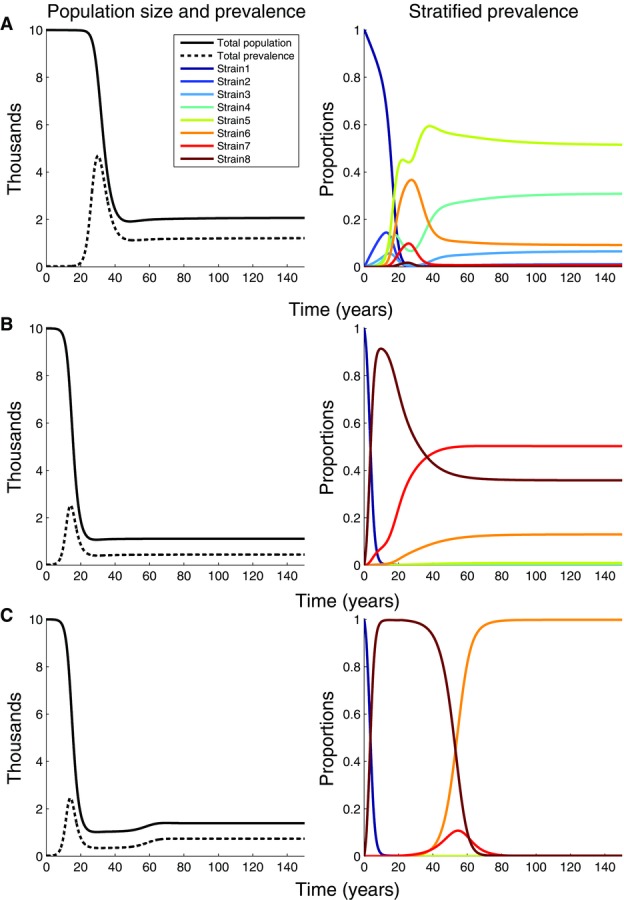
Epidemiological dynamics for the 8-strain model for different within-host fitness landscapes. In all numerical integrations the initial host population size is 10,000, the epidemic is initiated by a single individual infected with the least virulent strain, and all eight strains are equally transmissible (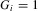 for all *i*). The first column shows the total host population size and the total prevalence of infection and the second column shows the proportion of infected individuals by infection type. (A) Flat within-host fitness landscape; all strains have the same within-host fitness. (B) Hill-climb fitness landscape where the fittest strain at the within-host level has a 2.5% fitness advantage over the least-fit strain (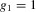 per day and 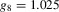 per day). (C) Rugged fitness landscape where the fittest strain at the within host level has a 2.5% fitness advantage over the least-fit strain (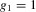 per day and 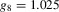 per day).

If we assume a flat within-host fitness landscape, the strain with the highest transmission potential (e.g., strain 5 in an eight strain scenario) rapidly becomes the most prevalent strain within the population, with the dynamics stabilizing after about 90 years from the start of the epidemic (Fig. 3A). This is in line with the conclusion reached by Shirreff et al. in their between-host model of HIV virulence evolution, even though their model did not include within-host evolution and instead included mutation at the time of transmission (Shirreff et al. 2011). For both the Shirreff model, and our model with a flat within-host fitness landscape, between-host processes drive the model and the dominant strain is the one that has the highest transmission potential. Once we assume a hill climb (Fig. 3B) or a rugged (Fig. 3C) within-host fitness landscape, more virulent strains dominate the population. Strains that are fittest at the within-host level outcompete strains that are fitter at the between-host level because of short-sighted evolution. The faster the within-host dynamics, the greater the influence within-host processes have on the epidemiological dynamics and the more myopic the short-sighted evolution. For example, where the within-host fitness landscape is a smooth hill-climb, the within-host dynamics are relatively fast (Fig. 2C) and a highly virulent strain dominates the population (Fig. 3B). Where the fitness landscape is more rugged, the within-host dynamics tend to be relatively slow (Fig. 2D) and a less virulent strain dominates the population (Fig. 3C). Allowing the composition of the viral population within the host at any particular time to influence the overall infectivity profile of the host (rather than only the viral strain initiating the infection) has only a minor effect on the epidemiological dynamics (not shown).

### EQUILIBRIA

**Figure 4:**
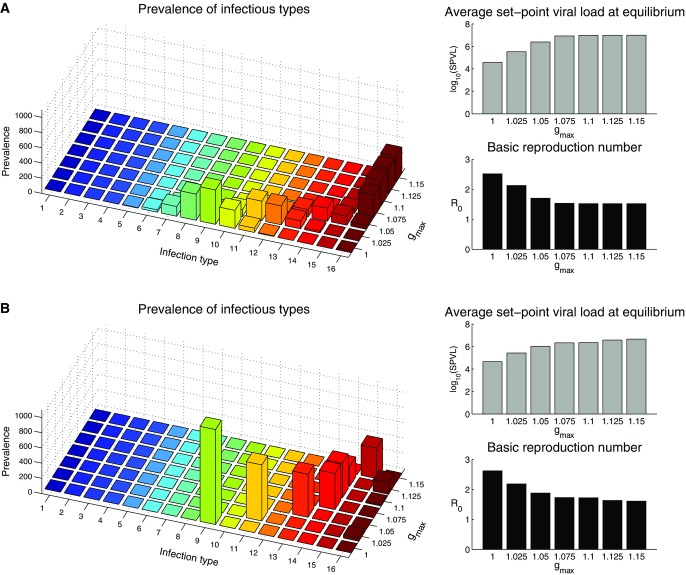
Equilibria for the 16-strain model for different within-host fitness landscapes and where all strains are equally transmissible (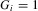 for all *i*). The left-hand column shows the prevalence of infections (z-axis, vertical) by infection type (x-axis) given the value of 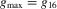 (y-axis). The right-hand column shows the mean SPVL at equilibrium (top) and *R*_0_ (bottom), given the intensity of within-host competition (i.e., the value of *g*_max_). Type-1 infections (i.e., initiated by the least virulent strain) are dark blue and type 16 infections are dark red. (A) Hill-climb within-host fitness landscape. (B) Rugged within-host fitness landscape. We can see that, as the intensity of within-host competition increases, more virulent strains dominate the population at the epidemiological level even though this reduces the fitness (*R*_0_) of the viral population.

To get a good understanding of the behavior of the model it is helpful to consider the system's equilibria. Assuming all strains are equally transmissible, increasing the difference in the within-host replication rate between the strains (i.e., increasing *g*_max_) results in more virulent strains dominating the population at the epidemiological scale (Fig. 4). This is because, as competition at the within-host level is intensified, within-host processes dominate over between-host processes, and as a result evolution becomes more short-sighted. This becomes evident if we consider the *R*_0_ of the viral population, which falls as within-host competition is intensified (Fig. 4A, B, bottom right panels). Having a more rugged fitness landscape (Fig. 4B) slows the within-host dynamics, meaning less virulent strains tend to dominate at the epidemiological level compared to when the within-host dynamics are much faster (Fig. 4A). Increasing the number of strains in the system slightly increases the *R*_0_ at equilibrium because adding more strains tends to slow the within-host dynamics (not shown). It is interesting to note that if we assume a hill-climb within-host fitness landscape, the equilibrium consists of multiple strains circulating between individuals (Fig. 4A), whereas if we assume a rugged fitness landscape, where all strains are equally likely to mutate into all other strains, strains are less likely to coexist (Fig. 4B). A clear understanding of these patterns can be derived by examination of the next generation matrix, *K* (not shown).

Recent evidence suggests that some strains of HIV-1 are more transmissible than others (Gnanakaran et al. 2011; Go et al. 2011), and we therefore consider the impact that allowing some strains to be inherently more transmissible than others has on the equilibria. We first consider the scenario where the relative transmissibilities of the strains are evenly distributed on a linear scale, and where the fittest strains at the within-host level are also the more transmissible:


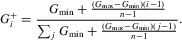
(10)

Throughout, 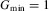 and 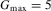, and the denominator is chosen to rescale the 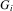
's to have mean 1.

**Figure 5:**
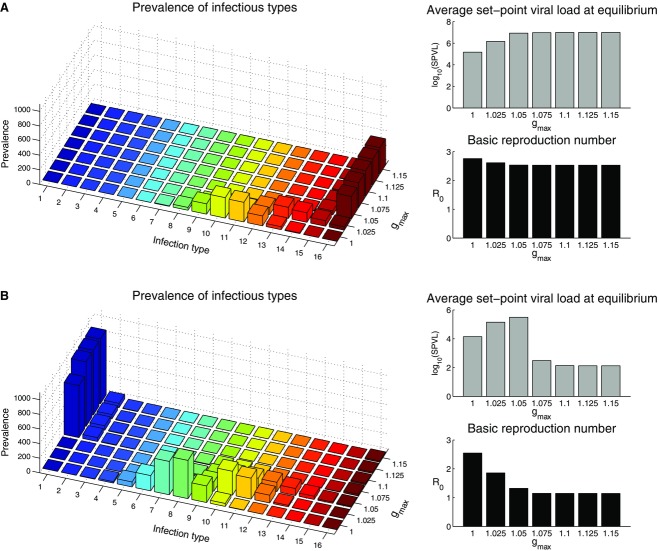
Equilibria for the 16-strain model with a hill-climb within-host fitness landscape and where strains are not equally transmissible. The left-hand column shows the prevalence of infections (z-axis, vertical) by infection type (x-axis) given the value of 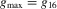 (y-axis). The right-hand column shows the mean SPVL at equilibrium (top) and *R*_0_ (bottom), given the intensity of with-host competition (i.e., the value of *g*_max_). Type-1 infections (i.e., initiated by the least virulent strain) are dark blue and type 16 infections are dark red. (A) The fittest strain at the within-host level is also the most transmissible (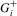, with 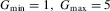, see equation (10)). (B) The least-fit strain at the within host level is the most transmissible (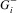, with 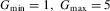, see equation (11)). Where we see the least-fit strain at the with-host level dominating at the epidemiological level, the equilibrium is unstable and the system exhibits oscillatory dynamics (see main text and Fig. 6).

As expected, infections tend to be initiated by more virulent strains than when all strains are equally transmissible (Fig. 5A), although the effect is fairly small (compare to Fig. 4A). The situation is not as clear-cut when the least-fit strain at the within-host level is most transmissible:


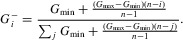
(11)

**Figure 6:**
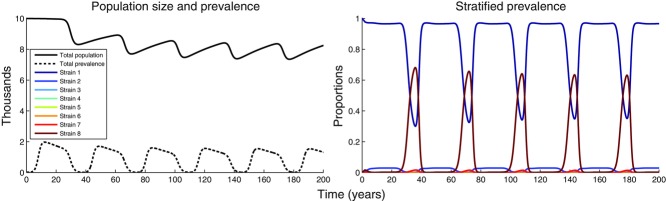
Epidemiological dynamics for the 8-strain model exhibiting oscillatory behavior. Here we assumed that the least-fit strain at the within host level is the most transmissible (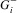, with 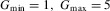, see equation (11)), and that 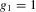
and 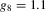. The system is run for 200 years to show that the oscillations are stable. A careful examination of the next generation matrix reveals that type-1 individuals (i.e., those infected by the least virulent strain) can alone drive a self-sustaining epidemic, because element *k*_1, 1_ of the next generation matrix is larger than 1. Other intermediate strains are also able to self-sustain but, irrespective of the starting conditions, sooner or later type-1 individuals dominate the epidemic because 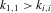 for all *i*. However, after their long asymptomatic period, the within-host dynamics will have selected for the most virulent strain (strain 8), which is transmitted during the AIDS phase. This generates a substantial number of type-8 individuals, which consume the susceptible population fast enough to stop the type-1 epidemic and quickly die because of their short infectious life and the inability to sustain an epidemic (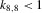). Further helped by the death of the type-1 individuals that reached the end of their infectious period, the overall prevalence collapses and, in a stochastic model, we would observe extinction of the viral population. However, in our deterministic model, the prevalence of type-1 individuals lingers at extremely low levels (the “attofox” problem of Mollison 1991) until the susceptible population grows enough to trigger another type-1 epidemic, and this generates the observed oscillations. Care has been put in choosing a sufficiently small time-step (*dt* = 0.005) for Euler's integration method not to yield negative results during the prevalence troughs.

For small values of *g*_max_, less virulent strains tend to dominate at the population level compared to when all strains are equally transmissible (compare Figs. 4A, 5B), as might be expected. However, for higher values of *g*_max_ (i.e., where the within-host dynamics are faster) we see a switch to the least virulent strain dominating new infections, with a small, but not negligible, number of new infections initiated by the most virulent strain. Numerical simulations (Fig. 6) reveal that this equilibrium is not attractive and the solution of the full dynamics exhibits stable oscillations between the most virulent and least virulent strain dominating the population. However, these oscillations are an unrealistic consequence of the deterministic nature of our model, often referred to as the “attofox” phenomenon (Mollison 1991; see Fig. 6 for a more detailed description of the dynamics in this unrealistic scenario). In a stochastic model, the viral population would be expected to go extinct after the first epidemic wave. This is because short-sighted evolution selects for the fittest within-host strain, but this strain is unable to drive a self-sustaining epidemic due to its inefficiency at transmitting between hosts.

## Conclusions/Discussion

We have presented a general framework allowing researchers to model multistrain within-between host models of pathogen evolution, and demonstrate the utility of the approach by studying the evolution of virulence in HIV infection. If there is little within-host evolution, because of limited competition between strains, a large number of competing strains, and/or a within-host adaptive landscape that is difficult to traverse, virulence will evolve toward an intermediate level that maximizes the transmission potential of infections and the number of hosts infected. However, if there are greater opportunities for within-host selection, virulence is expected to evolve toward a higher level even though this reduces the transmission potential of infections and the number of hosts infected.

Data suggest that the replicative capacity of HIV tends to slowly increase during the course of infections, but by a relatively modest amount compared to the variation in replicative capacities found at the population level (Troyer et al. 2005; Kouyos et al. 2011). If replicative capacities do increase by the small amount suggested in these studies, that is where the virulence of the strain an individual is infected with is similar to the virulence of the strain that individual tends to transmit, our model predicts that HIV will evolve a level of virulence very close to the level that will maximize the transmission potential of the virus. According to a recent meta-analysis, HIV virulence has increased over the past two decades, but the upward trend has plateaued off in the last few years (Herbeck et al. 2011). Because current levels of HIV virulence maximize the transmission potential of the virus (Fraser et al. 2007; Shirreff et al. 2011), we predict that HIV is unlikely to get much more virulent, if at all, in years to come.

Of course, it is interesting to wonder why the replicative capacity does not increase much more rapidly during the course of infection than it appears to do. One possibility is that the within host adaptive landscape is extremely large, rugged, and difficult to traverse (Kouyos et al. 2012). In such a situation, the within-host viral population will only be able to explore a small corner of the landscape and as a result between-host selection pressures will overshadow within-host evolution. In addition, the host immune system is likely to have a significant role. Although evidence suggests that the intrinsic replicative capacity (by which we mean the replicative capacity as measured *in vitro*) of the strain of HIV an individual is infected with determines SPVL (Quiñones-Mateu et al. 2000; Trkola et al. 2003; Daar et al. 2005; Joos et al. 2005; Kouyos et al. 2011), this might not be correlated with the ability of the virus to replicate in the face of an adaptive immune response, that is the realized replicative capacity of the virus during the course of infection. For example, strains harboring CTL escape mutations often have reduced *in vitro* replicative capacities, but will be under positive selection during the course of an infection (Mostowy et al. 2012). Consequently, although the intrinsic replicative capacity of the infecting viral strain will influence the transmission potential of the infection, this intrinsic replicative capacity might have only a small influence on the within-host dynamics. On a related issue, it is worth noting the small body of evidence showing that ancestral strains of HIV (i.e., those that initiate infections) are stored in memory T cells and then expressed and preferentially transmitted over strains circulating later in infection (Lythgoe and Fraser 2012; Redd et al. 2012a). Even if the reproduction rate of viruses increases substantially during the course of infection, transmission of ancestral strains would effectively by-pass this within-host evolution, ultimately favoring strains with the highest transmission potential.

As a final point, we reiterate that we have made some strong assumptions in this model. First, we have assumed here that all hosts are identical. However, we know that host genetic factors affect the ability of individuals to control HIV infection in terms of SPVL and duration of infection (Fellay et al. 2009). If the relative ranking of viral strains, in terms of their replicative capacity and virulence, tends to be the same in all hosts, we do not expect host heterogeneity to drastically alter our conclusions: we would still expect short-sighted within-host evolution to drive up virulence at the epidemiological level. However, if the situation is more complex and the relative ranking is different in different hosts, for example due to HLA heterogeneity, it is not immediately clear how this will affect the evolution of the viral population, and this is an important area of future study. Second, we have used a very idealized quasi-species approximation to model within-host viral dynamics, which is clearly an oversimplification; for example, new research is now enabling us to gain insight into the selection pressures faced by the virus at the very earliest stages of infection (Bar et al. 2012). Substituting the quasi-species model for a mechanistic within-host model that incorporates some of this complexity might provide useful insights, although the difficulty will be in keeping such models simple enough that they are still tractable. Finally, it is important to realize that the model presented here is deterministic. We would expect a stochastic version of the model to slow the rate of within-host evolution, thus tilting the balance toward strains that have a higher transmission potential, but it is not clear how strong this effect will be.

Predicting how virulence, or any other trait, is likely to evolve in any particular system is difficult because we have two levels of selection to consider, within hosts and between hosts, and often there are a large number of circulating strains (Shankarappa et al. 1999) that need to be considered. Here we have constructed a framework that allows researchers to link these two levels of selection and to accommodate a large number of pathogen strains. Although we have focused this framework on HIV, with a very simplistic model of within-host evolution, the modeling framework is general enough that it can be easily adapted to fit a broad range of situations in which one wants to model multiple strains circulating at the within- and between-host levels.

**Associate Editor: S. Remold**
